# Low- and High-Pressure Membrane Separation in the Production of Process Water for Coke Quenching

**DOI:** 10.3390/membranes11120937

**Published:** 2021-11-27

**Authors:** Anna Trusek, Maciej Wajsprych, Andrzej Noworyta

**Affiliations:** Division of Bioprocess, Micro- and Nanoengineering, Department of Chemistry, Wroclaw University of Science and Technology, Wybrzeze Wyspianskiego 27, 50-370 Wroclaw, Poland; maciej.wajsprych@gmail.com (M.W.); andrzej.noworyta@pwr.edu.pl (A.N.)

**Keywords:** coke-oven wastewater, membrane separation, water recovery, coke quenching

## Abstract

Although the time for operating mines and coking plants in many countries is coming to an end due to climate change, we must still ensure that the pollution generated by this source of the economy is minimized. Despite the several stages of treatment of the coke-oven effluent, completed with nitrification and denitrification processes preceding final sedimentation, the stream obtained does not meet the requirements of water for coke quenching. That is why the stream after biodegradation and sedimentation was treated on membrane units to ensure water reusing in the coking plant. As the subjected stream contained both solid and dissolved pollutants, a two-stage system was proposed: low- and high-pressure membrane filtration. Industrial modules were tested on pilot units operating under industrial plant conditions. In the case of the ultrafiltration process, all the tested ultrafiltration modules fulfilled the primary task. All of them separated almost completely the turbidities present in the stream, which would have disturbed the operation of the high-pressure plant. Considering the decrease in permeate flux and the possibility of cleaning, a PCI membrane made of PVDF tubes with a diameter of 12.5 mm and pore size of 20 μm was selected. Regarding the high-pressure membrane filtration, the reverse osmosis membrane was significantly better in the removal efficiency of both organic and inorganic dissolved substances. An operating pressure of 3 MPa was chosen for the system. Hence, membrane processes, which are not used as stand-alone treatment units for coke-oven effluents, function well as a final treatment stage.

## 1. Introduction

Mine operation is seen as one of the causes of climate change. Over 40 countries, including Poland, committed to abandoning coal at the COP26 climate conference in Glasgow in November 2021. However, it is not a process that will take place overnight. Additionally, the declaration was not signed by several countries that are the world’s most significant coal users. That is why today it remains necessary to create wise policies on exhaust gas treatment to protect the air and on wastewater management to reduce waste and ensure the water cycle in a plant.

Coking plants are a source of many hazardous substances produced during the coking of coal and the treatment of the resulting products [1,2]. The size of the wastewater stream, the multitude of present compounds, and their instability in time pose a problem for wastewater treatment. The required concentration values of individual compounds that can be discharged into surface waters are becoming increasingly stringent. They are most often referred to as phenolic wastewater due to the predominant content of this compound [3,4]. According to the place of generation, coking plant effluents can be divided into tar, gas cooling, benzol recovery, rectification, and tar processing. The primary chemical pollutants of coke oven effluents, apart from phenols, are organic compounds conventionally referred to as COD (chemical oxygen demand), ammonia, sulfides, and cyanides [5,6,7]. Their origin is mainly the installation of cooling and condensation of raw coke-oven gas leaving the coke-oven chambers.

A secondary condensate gas is obtained by repeated cooling. The collected condensate flows through a mechanical clarifier into the ammonia water tank. Further on, processes typical for coke-oven wastewater treatment plants take place. During extraction, the content of volatile phenols is reduced, and then the process of steam distillation for ammonia removal occurs. After tar removal, a coagulant containing iron ions (+2 and +3) is added. The iron ions in an alkaline environment react with sulfides and cyanides and bound cyanides, precipitating them as sludge [8,9]. Free cyanides and sulfides must be removed entirely (concentration below 0.1 ppm) because their presence in solution decreases the activity of the activated sludge used in the following (biological) stage of wastewater treatment. Slightly more lenient conditions are for the reduction of the bound cyanide. Their concentration in wastewater undergoing biological treatment cannot exceed 5 ppm.

Activated sludge is responsible for the process of nitrification and denitrification [10,11]. The carbon source used by activated sludge microorganisms is phenol and its derivatives present in the wastewater. The content of the main components of coke-oven wastewater before and after the integrated treatment described above is presented in Table 1.

The wastewater treated in this way is partly used in the water management of the coking plant. The quality of processes within the coking plant is strongly dependent on the quality of this water. The water used for coke quenching is the direct factor that ultimately determines the grade of the coke produced [13]. In practice, this is often utility water mixed with wastewater treated using the methods discussed above. The highest coke purity class can be obtained using water free of COD and desalinated, especially from chlorides and sulphates. For this reason, further purification of the final effluent to achieve water that is as free as possible from the components listed in Table 1 is a worthwhile task that leads to economic benefits.

A promising possibility to solve this problem is membrane processes application. The membrane techniques enable a deep purification of media containing suspended solids, molecules, and ions [14,15,16,17]. So far, membrane techniques have not found an industrial application in the treatment of coke-oven wastewater. Several reasons influence this state of affairs. First of all, coke-oven wastewater is a multi-component medium with variable and unstable compositions. In addition, the membranes are blocked by tarry substances, hence the need for their complete removal in the first stage of coke-oven effluent treatment and membrane regeneration during the process. Nevertheless, the stream obtained after chemical and microbiological purification, free from most impurities, can be subject to membrane separation.

In the literature regrading this subject, only a few papers can be found [18,19,20,21,22,23]. Kumar and Pal conducted a study on the filtration of coke-oven effluent using reverse osmosis and nanofiltration [18,19]. The experiments were carried out using flat modules and cross-flow at varying parameters, such as pressure, linear velocity, and pH of the feed. During reverse osmosis, COD, cyanides, phenols, and ammonium nitrogen were removed with a 96–98% efficiency, at a maximum permeate flux of 46 L/(m^2^·h). Better results, 99% compound removing, was obtained at a similar permeate flux value (45 L/(m^2^·h) during the nanofiltration process.

Kwiecinska and Rychlewska et al. [20,21] tested different types of polymeric, polyethersulfone membranes that differed in cut-off (equal to 20, 10, 5, and 3 kDa) and ceramic membranes with a zirconia active layer (5 and 8 kDa) to find the most efficient removal of complex cyanides and chemical oxygen demand (COD). The evaluation of processes was made on the basis of flux stability, fouling intensity, complex cyanides, and COD removal rates. The studies revealed that the ultrafiltration process enabled to remove complex cyanides up to 75%, whereas COD was decreased by 27%.

In experiments conducted by Jin et al. [22], nanofiltration and reverse osmosis were applied after a membrane bioreactor for biological treatment. The removals reached 82.5% (COD), 89.6% (BOD), 99.8% (ammonium nitrogen), 99.9% (phenol), 44.6% (total cyanide (T-CN)), 99.7% (thiocyanide (SCN−)), and 8.9% (fluoride) during the biological treatment stage. The nanofiltration–reverse osmosis system significantly reduced the parameters, including COD, T-CN, total nitrogen, fluoride, chloride ion, hardness, and conductivity, to a level suitable for industrial reuse, with a total water production ratio of 70.7%.

The combination of biodegradation with membrane processes (reverse osmosis) has also been confirmed by Pimple et al. [23]. The final rejection of cyanide in the RO permeate was above 90%, phenol above 95%, and total suspended solids was 100%. Thus, the permeate quality was found satisfactory and the process may be adopted at full-scale for treatment of coke-oven wastewater in the industry. 

This work aimed to select the membrane processes and the membranes used in them so that the stream leaving the installation (final permeate) fully meets the requirements of process water, and more precisely, the water for coke quenching. In this way, the water from the mine could be fully recycled, which would directly impact the management of water resources. Due to the scale of the application, it was important that laboratory-scale research could be transferred to an industrial scale. Hence, the study was carried out on a sizeable laboratory-scale apparatus and commercially available membranes for large-scale purposes.

## 2. Materials and Methods

### 2.1. Coke-Oven Wastewater

The effluent used in the experiments was delivered directly after the biological treatment and sedimentation process by the selected coke plant in Poland (the name protected by contract); its characteristics are presented in Table 2. There is a high concentration of sulfates and chlorides. These came from FeCl_3_ and Fe_2_(SO_4_)_3_, which are added during the preceding coagulation process. Moreover, the ratio of COD determined by the chromium method to BOD is high. It indicates that the organic content was well oxidized during biological treatment. The wastewater was brown, and the suspension tended to slow coagulation.

### 2.2. Membrane Modules and Installations

The modules used in this work were made in-house from commercially available membranes of varying selectivity. The module housings were made of PVC-U (Landefeld Druckluft und Hydraulik GmbH, Kassel-Industriepark, Germany). A number of membrane tubes was used in each module to ensure the required retentate flow rate (>4 m/s). The epoxy resin Epidian 5 (CMS Ltd., Gateshead, UK) and polyamide hardener Z1 (CMS Ltd., Gateshead, UK) at a ratio of 10:1 *w*/*w* were used to glue the membranes. The module housings were glued using PVC-U materials from Georg-Fischer Ltd. (Schaffhausen, Switzerland).

**Figure 1:**
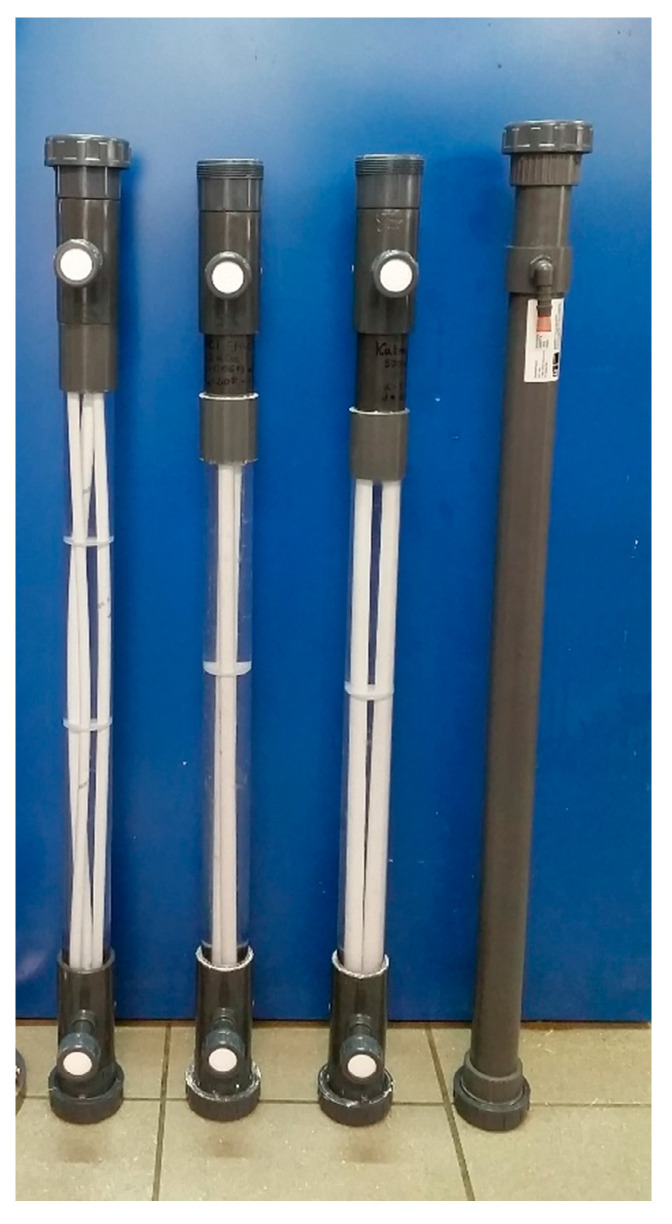
The modules used for low-pressure filtration.

The characteristics of the membranes and modules used in this study are presented in Table 3. Figure 1 presents the image of modules Nos. 1–4.

The membranes differ in their selectivity, the material they are made of, and the range of process parameters at which they can be used. An important issue is the pH and temperature range because they determine the possible ways of carrying out the chemical regeneration of the membrane. The limit values of the working parameters of the membranes used in this study are summarized in Table 4. Methods for regeneration of the membranes used were described in an earlier publication by the authors [24].

A Le Carbon Lorraine unit (Paris, France) and a Millipore installation (Burlington, MA, USA), presented in the Supplementary Materials, were used for the low- and high-pressure membrane filtration, respectively.

### 2.3. Analytical Methods

The process selectivity was estimated based on a few parameters such as COD, turbidity, color, alkalinity, and the concentration of selected cations and anions. All the analyses were carried out following the applicable procedures of the Polish Standardization Committee and are presented in Table 5. All the reagents used were of analytical grade and were purchased from Pol-Aura (Dywity, Poland).

## 3. Results

The treatment of coke-oven streams after biological treatment and sedimentation was divided into two stages: pre-treatment by low-pressure membrane filtration and deep treatment by high-pressure filtration.

### 3.1. Low-Pressure Membrane Filtration

Four different ultrafiltration membranes were used in the study. The hydraulic characteristics of these membranes were described in an earlier publication by the authors [24]. Based on these data, a pressure of 0.2 MPa was chosen for the study on contaminant separation.

**Figure 2:**
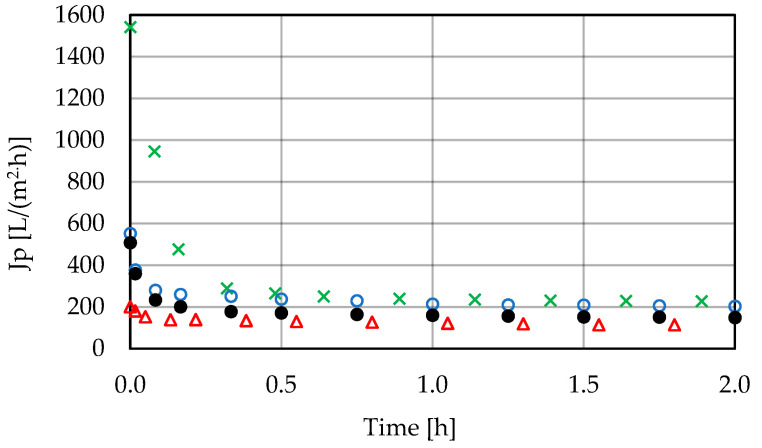
Change in permeate flux during low-pressure filtration (T = 25 °C, w = 4 m/s, ΔP = 0.2 MPa); ×, Berghof 0.03 mm; o, Katmaj 500 kDa; Δ, Burkert CUT 50 kDa; •, PCI 20 kDa. Points represent the average of five measurements.

**Figure 3:**
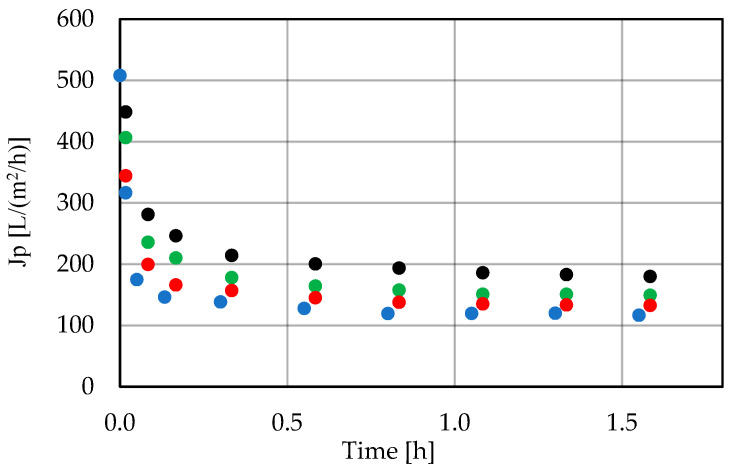
Change in permeate flux during low-pressure filtration for the PCI 20 kDa membrane (T = 25 °C, w =4 m/s, ΔP = 0.2 MPa) using the initial feed (•) and 2 (•), 4 (•), and 6 (•) times concentrated feeds. Points represent the average of five measurements.

A speedy decrease in the permeate flux was observed for each of them during the first few minutes, followed by a period of pseudo-stable operation of the system (Figure 2). The permeate flux obtained during this period was in the range of 120–210 L/(m^2^·h). The degree of feed concentration slightly affected the permeate flux change profile (example curves, for the PCI membrane, are presented in Figure 3).

Samples for analysis of the obtained permeates were taken in a pseudo-stationary state (after one hour). The composition of the feed varied from experiment to experiment, which is due to the variability in the coke-oven effluents. The permeate quality obtained for all the membranes tested was similar although the pore size varied considerably (Table 6).

### 3.2. High-Pressure Membrane Filtration

**Figure 4:**
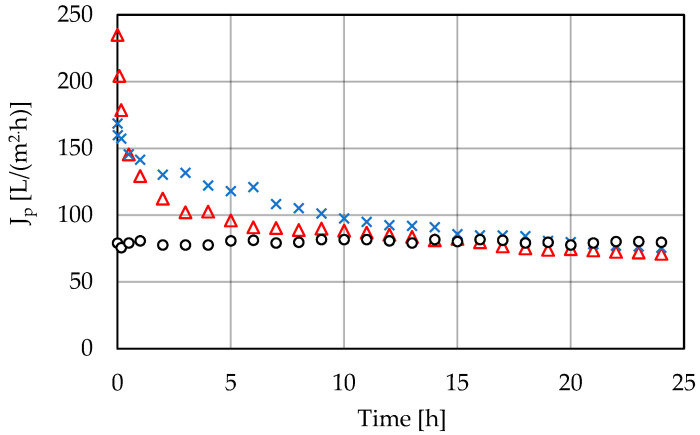
Permeate flux change during the nanofiltration process; PCI NF membrane, T = 25 °C (o, 1.0 MPa; ×, 2.0 MPa; Δ, 3.0 MPa). Points represent the average of five measurements.

**Figure 5:**
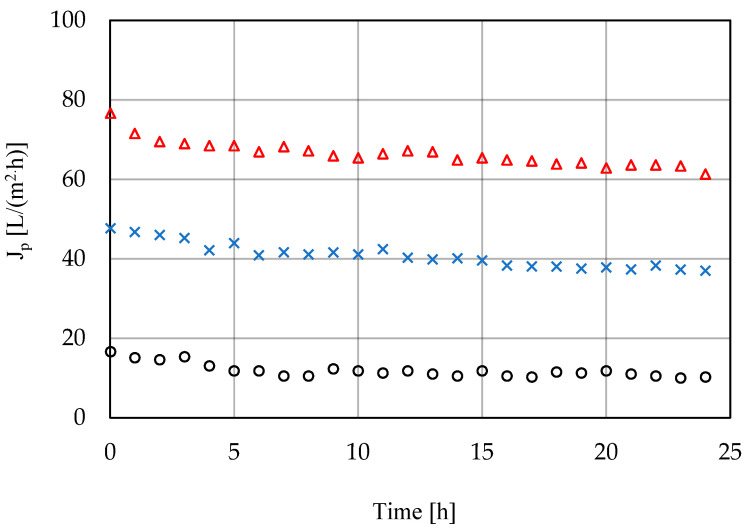
Permeate flux change during the reverse osmosis process; PCI RO membrane, T = 25 °C (o, 1.0 MPa; ×, 2.0 MPa; Δ, 3.0 MPa). Points represent the average of five measurements.

The high-pressure filtration process was carried out with polyamide membranes, distinguished by the manufacturer (PCI) as nanofiltration (75% CaCl_2_ retention) and reverse osmosis (99% CaCl_2_ retention). The feed in these processes was the permeate obtained during ultrafiltration on the PCI 20 μm membrane. High-pressure membrane filtration was carried out at transmembrane pressures of 1–3 MPa, at a temperature of 25 °C for a period of 24 h. The change in permeate flux over time is presented in Figure 4 and Figure 5.

**Figure 6:**
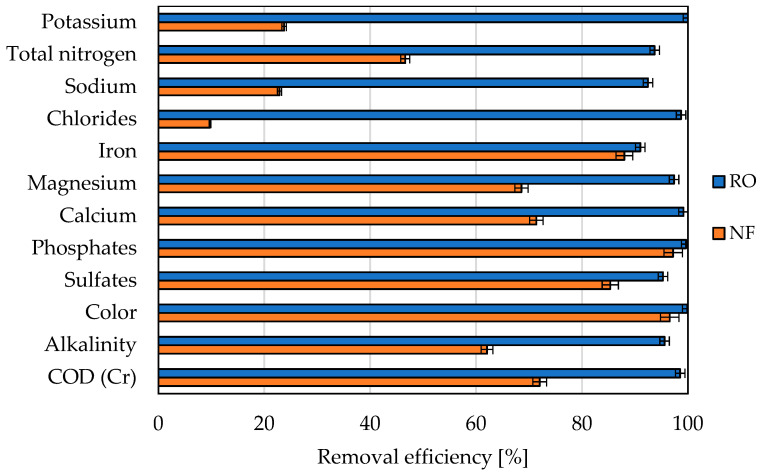
The removal efficiency of the selected compounds during high-pressure filtration; PCI NF membrane and PCI RO membrane, T = 25 °C, ΔP = 3.0 MPa.

The removal efficiency of the selected compounds during high-pressure filtration, expressed as the concentration of a given compound in the permeate to its concentration in the feed, is shown in Figure 6.

## 4. Discussion

Although the membranes used in ultrafiltration differed significantly in pore size (from 0.03 mm to 20 kDa), the decrease in permeate flux and separation capacity were similar for all membranes. The ultrafiltration process was mainly considered to remove solids (turbidity), which would cause unstable operation of the high-pressure filtration plant. The effect of eliminating other components was taken as an additional benefit. It was obtained at a relatively high level for color compounds (more than 50% removal) and oxygenation compounds (COD parameter higher than 20%). With the highest degree of turbidity removal in mind, two membranes can be selected: Berghof 0.03 µm and PCI 20 kDa. The high solids separation involving a membrane with a pore size of 0.03 μm is a surprising result. Particle size analysis (presented in the Supplementary Materials) performed with the Sald-2300 apparatus (Shimadzu, Kyoto, Japan) indicated that the size spread of the solid particles present in the coke-oven wastewater is between 0.027 and 0.063 μm, and the dominant fraction at about 0.04 μm. Analyzing the membrane material, PVDP was slower to block than PES. Both membranes indicated are made of PVDF. Due to the flow hydrodynamics and ease of cleaning, the larger-diameter tubes (20 kDa PCI membrane) appear to be a better choice. Independent studies developed a method for its regeneration after using in the treatment of mine wastewater [24].

The actual (final) separation took place during the high-pressure processes. The unfulfilled requirements after a biodegradation process for the process water for coke quenching mainly concerned COD and salination, especially coming from chlorides and sulphates. As expected, membrane separation using a reverse osmosis membrane was much more effective than separation using a nanofiltration membrane in terms of selectivity. The greatest differences were observed for monovalent ions, including chlorides, according to the theory of membrane processes and previous studies [25,26,27]. Removal of COD in the NF process was at a relatively low, unexpected level (approx. 73%). Since high flux drops were observed for the NF membrane at all pressures tested, in contrast to the separation for the RO membrane, the high energy expenditure, a widely recognized result due to the use of higher pressures for the reverse osmosis process, was in this case practically the same as for the NF membrane. At a pressure of 3 MPa, after 24 h, the permeate flux for both membranes was similar.

As the separation properties of the PCI RO membrane were significantly higher (for all compounds, the removal above 90%), this membrane is recommended for mine wastewater treatment. The content of all compounds is low enough that the water obtained after reverse osmosis can be directly used for coke quenching.

The use of retentates (waste streams) was not undertaken in the presented research, but it is crucial to consider in further studies. Especially, the retentate after the ultrafiltration process, which is rich in organic matter, should be managed following the principle of clean technologies. As it is a stream with a dominant water fraction, its treatment could start with hydrothermal treatment, as was described for another aqueous waste stream [28].

Different processes, with their advantages and limitations, regarding energy and material cycling for sustainable solid waste management are presented in many papers [29,30,31,32]. They can be the base to develop a management technology for solids coming from coke-oven wastewater.

## 5. Conclusions

The use of membrane processes fully closes the water cycle in the mine, which allows for a significant improvement in water management. Using two-stage membrane separation, process water within the parameters for direct use in coke quenching processes was obtained.

Based on the research, the stream obtained after biodegradation and sedimentation should be subjected first to ultrafiltration and then reverse osmosis. For ultrafiltration processes, it is recommended to work with the membrane PCI 20 kDa, at 0.2 MPa. Due to the substantial flux drop during filtration, frequent regeneration of this membrane is necessary. In previously described studies [24], cleaning with sponge balls was selected for the PCI membrane. To remove dissolved organic (and thus COD) and inorganic compounds, it is recommended to use the PCI RO (99% NaCl retention) membrane at 3 MPa. Membrane operation under these conditions is stable for at least one day, after which a short-term backflushing is recommended. The research carried out on industrial membranes significantly reduce the amount of verification testing needed on the target plant. Its design can be done based on the presented results.

## Figures and Tables

**Table 1:** Selected values for raw wastewater and after treatment with a final stage involving activated sludge [12].

Parameter	Raw Wastewater (mg/L)	After Treatment (mg/L)
COD	6446	<250
Phenols	1656	<0.1
Ammonium	4349	<10
Complex cyanides	20	<5
Thiocyanates	401	<10
Salinity	7946	<5623
Sulfides	67	<0.1

**Table 2:** The average composition of the stream after the biological treatment and sedimentation process from a coke plant (the name protected by contract) in Poland (data received from the company).

Parameter	Unit	Range	Mean Value
pH	pH	8.2–8.5	8.4
Conductivity	mS/cm	11.3–12.1	11.7
Total nitrogen	mg N/L	50.5	50.5
Organic nitrogen	mg N_org_/L	6.4	6.4
Ammonia	mg N_NH4_/L	0.7–32.7	16.7
Nitrate	mg N_NO3_/L	10.6–25.4	18.0
Nitrite	mg N_NO2_/L	0.8	0.8
Phosphates	mg P/L	4.1	4.1
Total phosphorus	mg P/L	4.2	4.2
Oxygen dissolved	mg O_2_/L	8.9	8.9
BOD	mg O_2_/L	8.3	8.3
COD (Mn)	mg O_2_/L	165.8	165.8
COD (Cr)	mg O_2_/L	398–795	617
Total hardness	mg CaCO_3_/L	339.3–857	598.1
Alkalinity	mg CaCO_3_/L	550–700	625
Turbidity	NTU	4.7–34.5	17.3
Sulfates	mg SO_4_/L	1663–1780.5	1721.8
Chlorides	mg Cl/L	3463–5150	4306.5
Sodium	mg Na/L	200.4–233.7	217.0
Potassium	mg K/L	8.9–11.6	10.3
Calcium	mg Ca/L	28–78.7	53.4
Magnesium	mg Mg/L	5.6–17.2	11.4
Manganese	mg Mn/L	0.1	0.1
Iron	mg Fe/L	1.9–5.1	3.5
Color	mg/L	300–1560	930
Total dry matter	mg/L	7730	7730
Mineral dry matter	mg/L	480	480
Organic dry matter	mg/L	7250	7250
TDS	mg/L	7540	7540
Mineral TDS	mg/L	365	365
Organic TDS	mg/L	7175	7175
Total suspension	mg/L	190	190
Mineral suspension	mg/L	115	115
Organic suspension	mg/L	75	75

**Table 3:** The parameters of the membrane modules used during this research.

No	Type	Producer	Material	Selectivity	Diameter (mm)	Length (mm)	No of Tubes	Area (m^2^)	Cross-Section Area (m^2^)
1	UF	Burkert CUT Ingelfingenm, Germany	PES	50 kDa	8.0	795	3	0.059	5.03 × 10^−5^
2	UF	PCI Hampshire, UK	PVDF	20 kDa	12.5	795	2	0.062	1.23 × 10^−4^
3	UF	Katmaj, Herford, Germany	PVDF	500 kDa	12.5	795	2	0.062	1.23 × 10^−4^
4	UF	Berghof, Leeuwarden, The Netherlands	PVDF	0.03 μm	8.0	950	13	0.310	5.03 × 10^−5^
5	NF	PCI Hampshire, UK	PA	75% CaCl_2_	12.5	300	2	0.024	1.23 × 10^−4^
6	RO	PCI Hampshire, UK	PA	99% NaCl	12.5	300	2	0.024	1.23 × 10^−4^

**Table 4:** The limit values of the working parameters of the membranes.

No	Type	Producer	Material	pH Range	Maximum Temperature (°C)	Maximum Pressure (MPa)
1	UF	Burkert CUT	PES	2–11	50	0.7
2	UF	PCI	PVDF	1.5–10.5	60	0.7
3	UF	Katmaj	PVDF	1.5–10.5	90	1.0
4	UF	Berghof	PVDF	2–10	40	0.6
5	NF	PCI	PA	1.5–9.5	60	6.0
6	RO	PCI	PA	1.5–12	80	6.4

**Table 5:** The methods for the selected parameters’ determination.

Parameter	Kind of Method	Procedure	Equipment
COD	Titration method	PN-ISO 6060:2006	Titrator Compact G20S, Mettler Toledo
Turbidity	Nephelometric method	PN-EN ISO 7027:2016	Turbidity Meter TB1000 Thermo Scientific
Color	Spectrophotometric method	PN-EN ISO 7887:2002	Spectrophotometer UV-1800 Shimadzu
Alkalinity	Titration method	PN-EN ISO 9963-1:2001	Titrator Compact G20S, Mettler Toledo
Total nitrogen	Kjedahl method	PN-EN 25663:2001	Titrator Compact G20S, Mettler Toledo
Calcium Iron Magnesium Potassium Sodium	Atomic Absorption Spectrometry		ASA ICE3000 Thermo Scientific
Chlorides	Titration method	PN-ISO 9297:1994	Titrator Compact G20S, Mettler Toledo
Phosphates	Spectrophotometric method	PN-ISO 6878/1:2006	Spectrophotometer UV-1800 Shimadzu
Sulfates	Gravimetric method	PN-ISO 9280:2002	Analytical balance AS 160.R2 Radwag

**Table 6:** Degree of removal of the selected components during low-pressure filtration (T = 25 °C, w = 4 m/s, ΔP = 0.2 MPa).

Parameter	Unit		Berghof 0.03 µm	Katmaj 500 kDa	CUT 50 kDa	PCI 20 kDa
COD	mg _O2_/L	Feed	479 ± 13	795 ± 16	540 ± 12	498 ± 12
		Permeate	289 ± 11	578 ± 13	367 ± 10	384 ± 13
	% removal		39.7 ± 2.4	27.3 ± 2.3	32.0 ± 1.9	22.9 ± 2.6
Turbidity	NTU	Feed	32.2 ± 1.41	12.6 ± 0.81	34.5 ± 2.36	18.6 ± 0.74
		Permeate	0.28 ± 0.08	0.38 ± 0.07	1.15 ± 0.11	0.23 ± 0.09
	% removal		99.1 ± 0.4	97.0 ± 0.48	96.7 ± 0.39	98.8 ± 0.68
Color	mg _Pt_/L	Feed	2050 ± 154	1560 ± 87	300 ± 22	1840 ± 102
		Permeate	736 ± 77	750 ± 64	150 ± 19	830 ± 82
	% removal		64.1 ± 3.8	51.9 ± 3.4	50.0 ± 2.7	54.9 ± 3.3
Iron	mg _Fe_/L	Feed	6.22 ± 0.43	1.93 ± 0.20	5.05 ± 0.36	2.7 ± 0.27
		Permeate	0.24 ± 0.04	0.150 ± 0.02	0.17 ± 0.02	0.14 ± 0.01
	% removal		96.1 ± 0.54	92.2 ± 0.33	96.6 ± 0.58	94.8 ± 0.81
Calcium	mg _Ca_/L	Feed	107.1 ± 3.64	100.5 ± 4.48	78.8 ± 2.33	94.7 ± 3.49
		Permeate	100 ± 2.99	95.7 ± 3.02	68.1 ± 2.14	91.6 ± 3.19
	% removal		6.6 ± 2.74	4.8 ± 2.29	13.6 ± 4.28	3.3 ± 1.86

## Data Availability

Not applicable.

## Supplementary Materials

The following are available online at https://www.mdpi.com/article/10.3390/membranes11120937/s1, Figure S1. The pilot installation of Le Carbon Lorraine for low pressure driven membrane filtration: 1—feed water tank, 2—membrane module, 3—recirculation pump, 4—valve, 5—backflushing system, 6—compressor, 7—weight. Figure S2. The pilot installation of Millipore for high pressure membrane filtration; 1—membrane module, 2—membrane pump, 3—tank. Figure S3. The size of solids in coke oven wastewater analyzed with Sald 2300 (Shimadzu, Kyoto, Japan).
