# Supplementary material for: Low- and High-Pressure Membrane Separation in the Production of Process Water for Coke Quenching
Source: Membranes (Basel). 2021 Nov 27;11(12):937. doi: 10.3390/membranes11120937 (PMC8705688; doi:10.3390/membranes11120937)
Supplement: Supplementary file 1 [file membranes-11-00937-s001.zip › membranes-1474928-Figure S3.pdf]

Median D	0.038	Mean V	0.039
Modal D	0.035	Std Dev	0.077

25.000%D(μm)	50.000%D(μm)	75.000%D(μm)	0.000%D(μm)	0.000%D(μm)	0.000%D(μm)	0.000%D(μm)	0.000%D(μm)	0.000%D(μm)
0.045	0.038	0.034	0.000	0.000	0.000	0.000	0.000	0.000

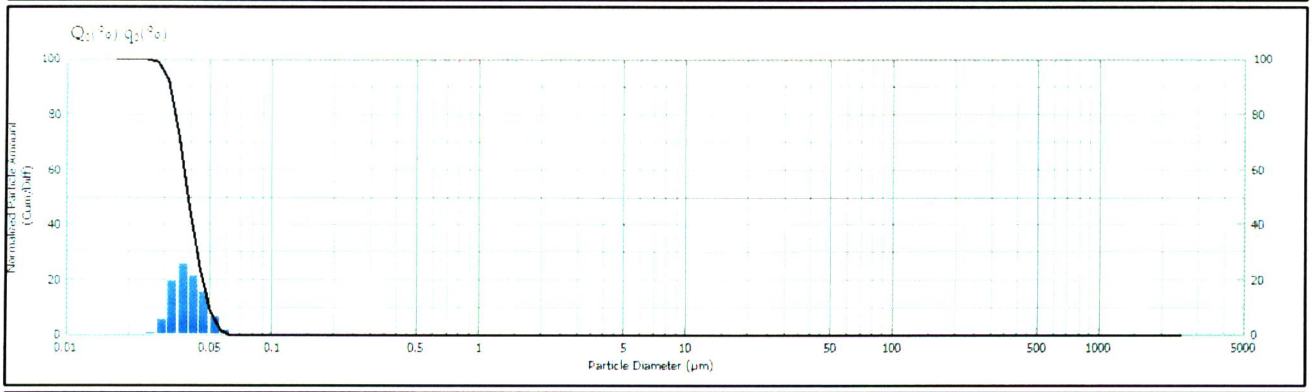

	Diam x(μm)	Cum% Q0(%)	Diff% q0(%)		Diam x(μm)	Cum% Q0(%)	Diff% q0(%)		Diam x(μm)	Cum% Q0(%)	Diff% q0(%)		Diam x(μm)	Cum% Q0(%)	Diff% q0(%)
1	2500.000	0.000	0.000	27	113.342	0.000	0.000	53	5.139	0.000	0.000	79	0.233	0.000	0.000
2	2219.551	0.000	0.000	28	100.628	0.000	0.000	54	4.562	0.000	0.000	80	0.207	0.000	0.000
3	1970.562	0.000	0.000	29	89.339	0.000	0.000	55	4.050	0.000	0.000	81	0.184	0.000	0.000
4	1749.505	0.000	0.000	30	79.317	0.000	0.000	56	3.596	0.000	0.000	82	0.163	0.000	0.000
5	1553.246	0.000	0.000	31	70.419	0.000	0.000	57	3.193	0.000	0.000	83	0.145	0.000	0.000
6	1379.004	0.000	0.000	32	62.520	0.000	0.000	58	2.834	0.000	0.000	84	0.129	0.000	0.000
7	1224.308	0.000	0.000	33	55.506	0.000	0.000	59	2.516	0.000	0.000	85	0.114	0.000	0.000
8	1086.965	0.000	0.000	34	49.280	0.000	0.000	60	2.234	0.000	0.000	86	0.101	0.000	0.000
9	965.030	0.000	0.000	35	43.752	0.000	0.000	61	1.984	0.000	0.000	87	0.090	0.000	0.000
10	856.773	0.000	0.000	36	38.843	0.000	0.000	62	1.761	0.000	0.000	88	0.080	0.000	0.000
11	760.661	0.000	0.000	37	34.486	0.000	0.000	63	1.563	0.000	0.000	89	0.071	0.000	0.009
12	675.330	0.000	0.000	38	30.617	0.000	0.000	64	1.388	0.000	0.000	90	0.063	0.009	1.967
13	599.572	0.000	0.000	39	27.183	0.000	0.000	65	1.232	0.000	0.000	91	0.056	1.976	7.000
14	532.312	0.000	0.000	40	24.133	0.000	0.000	66	1.094	0.000	0.000	92	0.050	8.976	16.038
15	472.597	0.000	0.000	41	21.426	0.000	0.000	67	0.971	0.000	0.000	93	0.044	25.014	21.570
16	419.582	0.000	0.000	42	19.023	0.000	0.000	68	0.862	0.000	0.000	94	0.039	46.583	26.063
17	372.513	0.000	0.000	43	16.889	0.000	0.000	69	0.766	0.000	0.000	95	0.035	72.647	20.006
18	330.725	0.000	0.000	44	14.994	0.000	0.000	70	0.680	0.000	0.000	96	0.031	92.652	6.341
19	293.624	0.000	0.000	45	13.312	0.000	0.000	71	0.604	0.000	0.000	97	0.027	98.993	1.007
20	260.685	0.000	0.000	46	11.819	0.000	0.000	72	0.536	0.000	0.000	98	0.024	100.000	0.000
21	231.442	0.000	0.000	47	10.493	0.000	0.000	73	0.476	0.000	0.000	99	0.022	100.000	0.000
22	205.479	0.000	0.000	48	9.316	0.000	0.000	74	0.422	0.000	0.000	100	0.019	100.000	0.000
23	182.428	0.000	0.000	49	8.271	0.000	0.000	75	0.375	0.000	0.000	101	0.017	100.000	0.000
24	161.963	0.000	0.000	50	7.343	0.000	0.000	76	0.333	0.000	0.000				
25	143.794	0.000	0.000	51	6.519	0.000	0.000	77	0.296	0.000	0.000				
26	127.664	0.000	0.000	52	5.788	0.000	0.000	78	0.262	0.000	0.000				

Condition File Name			
Ref Index	1.60-1.00i		
Decentralized Solvent		Dispersing Agent	
Denst of Disper Agent		Dispersion Method	
Stir Speed	Fast		
Sample Preparation			
Distribution Function	None	Smoothing Level	0
Select Emulation Table		Data Shift	0
Sequence File Name			
