# Supplementary material for: Low- and High-Pressure Membrane Separation in the Production of Process Water for Coke Quenching
Source: Membranes (Basel). 2021 Nov 27;11(12):937. doi: 10.3390/membranes11120937 (PMC8705688; doi:10.3390/membranes11120937)
Supplement: Supplementary file 1 [file membranes-11-00937-s001.zip › membranes-1474928-supplementary.pdf]

membranes

MDPI

Article

Low- and High-Pressure Membrane Separation in the Production of Process Water for Coke Quenching

Anna Trusek [1,*], Maciej Wajsprych [1] and Andrzej Noworyta [1]

[1] Division of Bioprocess, Department of Chemistry, Wroclaw University of Science and Technology, Micro- and Nanoengineering, Wybrzeze Wyspianskiego 27, 50-370 Wroclaw, Poland; maciej.wajsprych@gmail.com (M.W.); andrzej.noworyta@pwr.edu.pl (A.N.)

* Correspondence: anna.trusek@pwr.edu.pl; Tel.: +48-71-320-2653

Citation: Trusek, A.; Wajsprych, M.; Noworyta, A. Low- and High-Pressure Membrane Separation in the Production of Process Water for Coke Quenching. *Membranes* **2021**, *11*, 937. https://doi.org/10.3390/membranes11120937

Academic Editor: Marek Gryta

Received: 6 November 2021
Accepted: 25 November 2021
Published: 27 November 2021

Publisher's Note: MDPI stays neutral with regard to jurisdictional claims in published maps and institutional affiliations.

Copyright: © 2021 by the authors. Licensee MDPI, Basel, Switzerland. This article is an open access article distributed under the terms and conditions of the Creative Commons Attribution (CC BY) license (https://creativecommons.org/licenses/by/4.0/).

Supplementary Materials

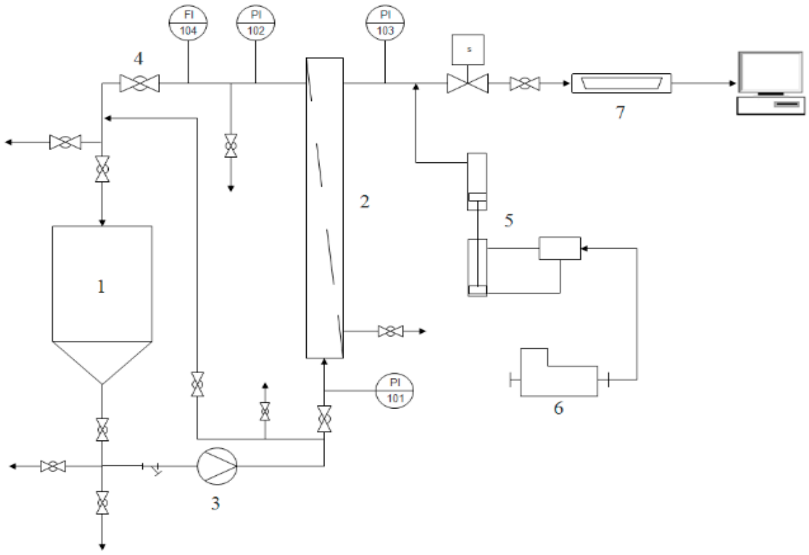

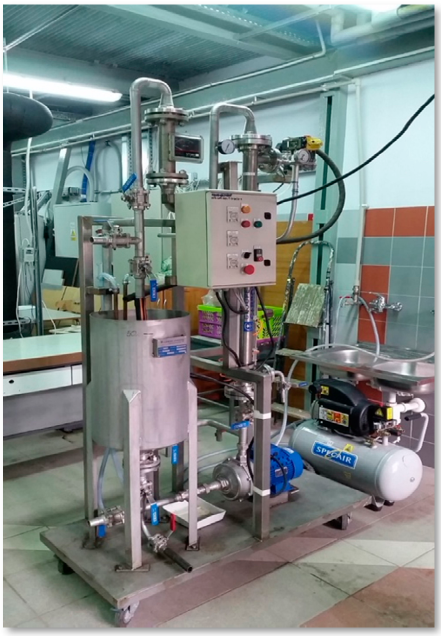

Figure S1. The pilot installation of Le Carbon Lorraine for low pressure driven membrane filtration: 1- feed water tank, 2 – membrane module, 3 – recirculation pump, 4 – valve, 5 – backflushing system, 6 – compressor, 7 – weight.

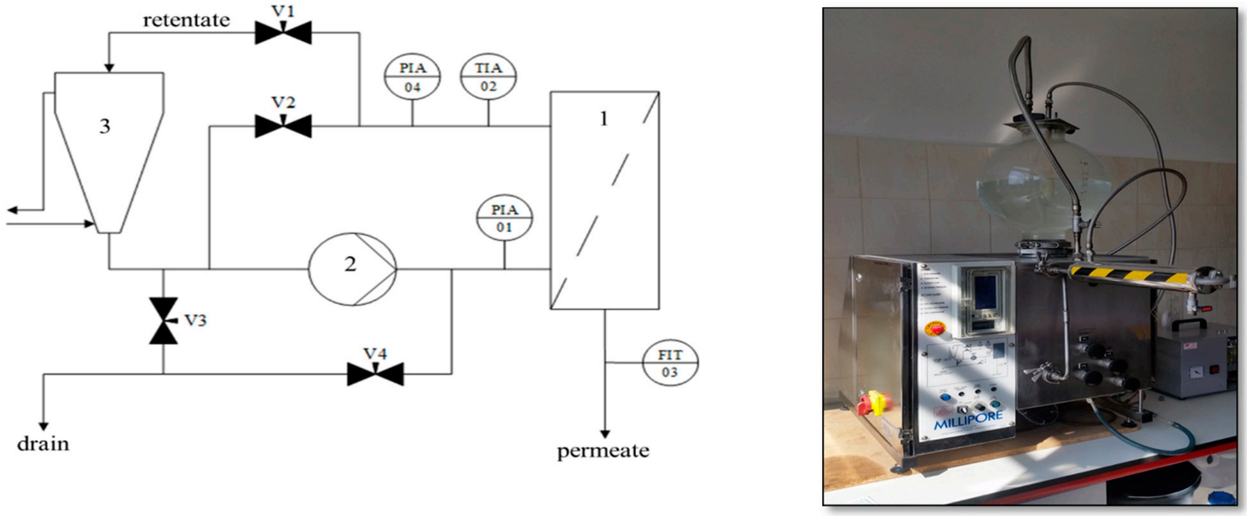

Figure S2. The pilot installation of Millipore for high pressure membrane filtration; 1 – membrane module, 2 – membrane pump, 3 – tank.
